# Assessing Smoking Habits, Attitudes, Knowledge, and Needs among University Students at the University of Milan, Italy

**DOI:** 10.3390/ijerph191912527

**Published:** 2022-09-30

**Authors:** Laura Campo, Silvia Lumia, Silvia Fustinoni

**Affiliations:** 1 EPIGET—Epidemiology, Epigenetics, and Toxicology Lab, Department of Clinical Sciences and Community Health, Università degli Studi di Milano, 20122 Milano, Italy; 2 Environmental and Industrial Toxicology Unit, Fondazione IRCCS Ca’ Granda Ospedale Maggiore Policlinico, 20122 Milan, Italy

**Keywords:** tobacco smoke, e-cigarette, heated tobacco products, passive smoke, questionnaire, university students

## Abstract

Background: College campuses and universities are valuable settings for smoking prevention programs targeting young adults. Aim: To investigate smoking habits, electronic cigarette (e-cig) and heated tobacco product (HTP) use, exposure to passive smoke, compliance with smoking bans on campus, attitudes toward the anti-smoking policies, and educational needs among students at the University of Milan, Italy. Methods: A validated questionnaire was web-submitted to 64,801 students in the period May–July 2021. For each item, the frequency was calculated and χ^2^ test with Bonferroni correction was used to compare differences among the 10 faculties of the University. Results: 7162 students participated in the survey, while 6605 questionnaires were included in this report (62% female, 84% aged 18–25 years). Sixty-four percent of participants were never smokers, 19% were smokers, 2.8% were e-cig or HTP users, 3.7% were dual smokers, 10% were former smokers, and 66% reported routinely spending free time with smokers. Almost all students were aware of the dangers of active and passive smoking of cigarettes, while about 20% did not have an opinion on the dangers of e-cigs/HTPs. Only 49% were aware of the smoking ban in the outdoor areas of the university. Students from the faculties of Law and Political, Economic, and Social Sciences smoked more frequently and were more frequently exposed to passive smoke than other students. Medicine students were the most aware of the dangers of passive smoking and using e-cigs/HTPs. Conclusions: This is the first study in Italy involving the entire student population of a university and highlighting differences among faculties in terms of active and passive smoking and opinions. The results suggest that prevention campaigns addressed to students should consider their specific study curricula and give information tailored to the different educational needs to efficiently support health promotion.

## 1. Introduction

Tobacco smoking represents one of the greatest public health problems worldwide as it is one of the major risk factors in the development of neoplastic, cardiovascular, and respiratory diseases. Globally, smoking accounts for about 8.7 million deaths/year [1], while in Italy, about 80,000 deaths/year were estimated [2,3]. A relevant issue is the diffusion of new nicotine-containing products, such as heated tobacco products (HTP), electronic cigarettes (e-cig), and other emerging products (i.e., hookah, smokeless tobacco, and dissolvable tobacco) particularly popular among the young population [4,5,6].

Under the frame of the WHO Tobacco Control Convention [7], several actions have been implemented in the last years in Italy, which resulted in a decrease in smoking prevalence, passing from 30% in 2001 to 22% in 2019 [2]. Legislative actions have been undertaken to address the issue of smoking among young people, among which is the specific ban on smoking on the external premises of schools and colleges [8]. However, smoking prevalence in Italy is still one of the highest in Europe, as about 19% of 15- to 16-year-old students are daily tobacco smokers, and as many as 30% report past 30-day smoking [9].

While smoking initiation generally occurs before age 18, young adulthood is the period during which smoking develops into regular use and nicotine dependence [10]. The main reason for this evolution is that, in this particular period of life, young adults face several changes, among which are a higher grade of independence and, for many, also leaving home. Therefore, young adults should be a primary target for smoking prevention and cessation programs [11]. On the contrary, evidence exists that the tobacco industry specifically markets to young adults [10], as is observed also for new tobacco products [12].

College campuses, and universities in general, are regarded as valuable settings for primary and secondary smoking prevention, as well as smoking cessation efforts targeting young adults [11], and some evidence exists that the implementation of smoke-control policies reduces smoking prevalence among students and staff, exposure to passive smoke, and cigarette butt littering [13,14,15,16]. Different types of smoking policies may be implemented in universities and campuses, from the simplest, including the restriction of smoking outdoors, to more advanced policies, including the total ban on all tobacco products. However, multi-level control programs including policy, education, and cessation aid are considered more effective in reducing smoking prevalence and passive smoke exposure.

In this context, the University of Milan (Italy), one of the biggest universities in Italy and known as “La Statale”, implemented a new regulation in 2019 banning all tobacco products in indoor and outdoor areas. However, enforcement difficulties were observed, probably due to the lack of communication and engagement on these issues and to the physical size and location of “La Statale”, which includes several buildings dedicated to 10 faculties, located in different areas of the city and with peculiar characteristics. To investigate the possible areas of intervention in anti-smoking policies, the University of Milan launched the project “La Statale Smoke-free” in the year 2020. We present here the result of the first step of the project, a web survey submitted to students to investigate their smoking habits, exposure to passive smoke, compliance with smoking bans on campus, and attitudes toward anti-smoking policies. Emphasis is given to differences possibly arising from the faculties of enrollment of students to obtain a profile of smoking behaviors useful for implementing new prevention campaigns and tailored actions for the anti-smoking policy. For this survey, a questionnaire previously validated and pilot-tested was used [17].

## 2. Materials and Methods

### 2.1. Study Population

The University of Milan is one of Italy’s youngest university institutions, as it was founded in 1924, and one of Italy’s biggest universities, as it hosts about 64,000 students. The University offers a multidisciplinary educational program, including 75 bachelor’s or single-cycle degrees (3- and 5- or 6-year courses, respectively), 66 master’s degrees (2-year courses), 34 doctoral programs (Ph.D., 3 years), 65 postgraduate schools (2–5 years), and 75 second level vocational master’s programs (www.unimi.it, (accessed on 7 September 2022)). Teaching activities are organized by 33 Departments and coordinated by 10 Faculties, including Law (Law), Political, Economic and Social Sciences (PESS), Humanities (Hum), Medicine (Med), Pharmacy (Pha), Science and Technology (STE), Agricultural and Food Sciences (Agr), Veterinary Medicine (Vet), School of Language Mediation and Intercultural Communication (LMIC), and Exercise and Sports Sciences (Sport).

In the academic year 2020–2021, the students enrolled in bachelor’s and master’s degree courses were 64,801, of which 38,568 were females (59.5%), and 26,233 (40.5%) were males (target students).

On 31 May 2021, an invitation letter, signed by the Rector of the University of Milan and containing the link to access the consent form and the questionnaire to assess smoking habits, was emailed to target students using their institutional e-mail address. In the following weeks, the students received four reminders, respectively on 14 and 28 June and on 12 and 28 July 2021. The survey was closed on 31 July 2021. The campaign was also promoted on the institutional website.

Once they received the mail, the students could express their informed consent and their will to participate in the study by clicking on the appropriate link. Only after giving their consent, students had access to the questionnaire. A copy of the informed consent was accessible to the participants by connecting to a dedicated link. Students could complete the questionnaire even only partially, and then resume it to complete or modify the responses already given. It was requested to answer each question to continue with the questionnaire. Once the final version had been sent, the answer was registered, and it was not possible to open the link again. Both an Italian and an English version of the questionnaire were available to students.

The study was approved by the ethics committee of the University of Milan [project code 82/20].

### 2.2. Questionnaire for the Assessment of Smoking Habits, Attitudes, Knowledge, and Needs

The questionnaire used for the study has been developed and validated as previously described [17]. The questionnaire included seven sections and 92 items: demographics (A, 6 items), current smoking of traditional tobacco cigarettes (B, 12 items plus 6 items from Fagerström Test to evaluate the nicotine dependence), past use of traditional tobacco cigarettes (C, 7 items), current or past use of electronic cigarettes or HTP products (D, 21 items), passive smoke exposure (E, 6 items), awareness of health issue related to smoking (F, 15 items plus 8 items related to the role of health professionals in helping patients to quit smoking derived from the WHO Global Health Professions Student Survey (GHPSS) [18], and knowledge and attitudes towards Italian smoking legislation, and educational needs (G, 11 items).

The first section of the questionnaire included a smoking status declaration defined as follows: never smoker (i.e., never smoked or have smoked less than 100 cigarettes in life), past smoker (current nonsmoker, but a person who has smoked more than 100 cigarettes in life), current smoker of traditional tobacco cigarettes exclusively, the current user of electronic cigarette or HTP products exclusively, current dual users (both traditional and e-cigs or HTPs). Based on this smoking declaration, participants had access to different sections of the questionnaire.

### 2.3. Data Analysis

Before statistical processing, collected data were anonymized by the University Teaching and Learning Innovation and Multimedia Technology Centre (CTU), by eliminating the link between the answers provided by the students and their identity. This was necessary because students accessed the questionnaire through their personal e-mail addresses. The anonymized data were provided by CTU as a Microsoft Excel file. Statistical analysis was performed using the IBM SPSS software package (ver. 27 for Windows, SPSS Statistics, IBM Italia, Segrate, Milano, Italy). For each item (categorical variable), the frequency was calculated for each response, while the mean and interquartile range were calculated for continuous variables. The χ^2^ test with Bonferroni correction was used to compare differences among faculties, with *p* ≤ 0.05 considered statistically significant. As the questionnaire could be completed even partially, only the questionnaires containing at least the smoking declaration were considered valid for the statistical analysis and were included in this report.

## 3. Results

### 3.1. Study Participants

**Figure 1:**
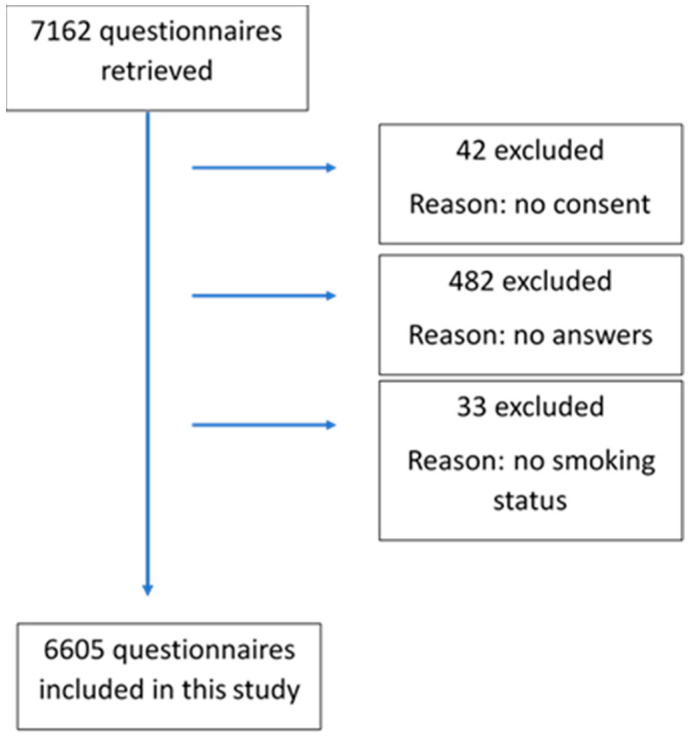
Flow chart of the inclusion process of participants.

The questionnaire was accessed by 7162 students, that is 11.0% of the eligible student population. The first call was answered by 2592 students (36.2% of the participants), the second by 2494 students (34.8%), the third by 943 students (13.2%), the fourth by 766 students (10.7%), and the last by 367 students (5.1%). Out of 7162 participants, 42 were excluded because they did not express their consent to participate in the study, 482 because they did not answer any questions, and 33 because they did not declare their smoking status. Therefore, 6605 students were eligible for this study (participation rate of 10.2%) (Figure 1).

Table 1 shows the main characteristics of the study participants in comparison with the student population of the University of Milan. The study participants were mainly female (62.1%), in the 18–25 years age range (84.1%), and from the Hum (21.9%) and STE (20.5%) Faculty. A minimal percentage of students declared to be intersex (0.3%) or did not declare their sex (1.1%). Median (5th–95th percentile) age was 22 (19–32) years (range 18–71 years). The study participants’ characteristics were very similar to those of the eligible student population as regards sex (female 62.1 vs. 59.5% in participants and student population, respectively) and age (84.1 vs. 78.9% in the age-range 18–25 years). Moreover, for each faculty, the percentage of students enrolled was similar to the percentage of students participating to the survey, even if students from the Law faculty were somewhat less represented among participants (6.7% participating vs. 10.5% enrolled in the faculty), while students from STE were slightly over-represented (20.5 vs. 15.4%) (Table 1). The participation rate ranged from 6.5 (Law) to 13.6% (STE) (mean 10.2%).

### 3.2. Active Smoking Habits

Table 2 reports smoking habits among all study participants and students stratified by sex, age, and faculty. Most students (63.8%) classified themselves as never smokers, 10.4% as former smokers, 19.3% as current smokers of traditional cigarettes exclusively, 2.8% as current users of e-cigs or HTPs only, and 3.7% as dual smokers. Therefore, 25.8% of students were found to be smokers of any product.

Smokers of tobacco cigarettes were prevalently males (22.3% vs. 17.3% of females, *p* < 0.001), while no sex differences were evident among users of e-cigs/HTPs (2.9% vs. 2.8%, in males and females, respectively) or among dual users (3.9% vs. 3.5%). The prevalence of both never smokers and actual smokers was different among the faculties (*p* < 0.001): in particular, the faculties of Law, PESS, and Hum had the lowest prevalence of never smokers and the highest prevalence of currently exclusive cigarette smokers. For Med, the prevalence of current smokers was 15%, but notably, differences were found among the different courses: the prevalence was the lowest among students from the 6-year single cycle Medicine degree (10%), while it was the highest among students from the 3-year Nursing courses (24.1%) (data not shown).

For e-cigs and HTPs exclusively users, no significant difference was found among the faculties, even if the highest prevalence of exclusively vapers was found among Law students, while the lowest among Sports students.

Overall, considering also dual smokers, the faculties with the highest prevalence of current smokers of any products were PESS (35.1%), Law (33.6%), and Humanities (30.2%) (*p* < 0.001).

When considering differences by sex among faculties, male students from PESS and Hum smoked with a higher prevalence than male students from Med and STE (32.5 and 41.5% vs. 17.8 and 24.5%, respectively, *p* < 0.05), while female students from Law (33.4%), PESS (30.9%), and Hum (28.0%) smoked with a higher prevalence than female students from Med (19.2%), Pha (16.6%), STE (17.6%), and Agr (15.4%) (*p* < 0.05).

Also considering differences by age groups among faculties, students in the age group 18–25 years from Law (38.0%), PESS (36.2%), and Hum (31.1%) smoked with a higher prevalence than students from the other faculties (range 18.9% for Med—25.9% for LMIC, *p* < 0.05) in the same age group. No differences were observed among students in the age group ≥26 years from different faculties.

### 3.3. Active Smoking of Traditional Tobacco Cigarettes

Table 3 and Table S1B report the results about traditional cigarette smoking and intention to quit among students (including exclusively and dual smokers). Most smokers (77.3%) declared to smoke daily, and less than 10 cigarettes/day (71.5%), with no significant differences among faculties. The age of initiation was 16 years old (range 9–29), with peer pressure (53.3%) and pleasure (53.5%) as the main reasons to start smoking.

At the Fagerström test, 75.4% of smokers resulted to have mild nicotine addiction, 16.4% a moderate, 7.3% strong, and 1.9% very strong addiction. Most smokers (59.4%) tried to quit smoking at least once, remaining abstinent for less than 6 months (76.1%), with no significant differences among faculties. Only 36.6% of smokers reported being advised by a doctor to quit smoking, with the prevalence among Med students higher than PESS, Hum, Pha, and STE students (*p* < 0.001). Moreover, only 21.3% were planning to quit smoking in the next six months, with no difference among faculties.

Most smokers smoked only outdoors (59.7%), but a relevant percentage (39.6%) reported smoking both outdoors and indoors. While on campus, most smokers (85.2%) reported to smoke only outdoors, and 25.8% only in smoking areas.

Among former traditional cigarette smokers, the great majority (92.3%) reported they had quit smoking without help, and the main reasons for quitting were concerns for their own health (70.9%) or to save money (35.8%) (Supplementary Table S1C).

### 3.4. Electronic Cigarettes or HTP Use

A relatively small percentage of students reported current e-cig (3.6%) or HTP (4.4%) use (both exclusively and dual users), but as much as 16.8% for e-cigs, and 9.3% for HTPs, reported ever use, with differences among faculties (*p* < 0.001). In particular, for e-cigs, a higher frequency of ever users was found in students from Law (23.8%) than Med (12%), Pha (14.9%), and STE (15.1%) faculty, and students from PESS (20.1%), Hum (17.4%), and Agr (19.1%) than Med (results not shown). Similarly, for HTP, a higher frequency of ever users was found in students from Law (16.3%) than Hum (8.7%), Med (8.0%), Pha (8.5%), STE (5.9%), and Agr (7.1%) faculty, and in students from PESS (14.0%) than Hum, Med, STE, and Agr (results not shown).

Among current users, most had been using e-cigs (44.3%) or HTPs (39.8%) for more than one year (Supplementary Table S1D). More than 73% of students used e-cigs and HTPs both outdoors and indoors, in contrast with traditional cigarettes (40%) that were mostly used only outdoors. While on campus, most students (>56%) used these products only outdoors, even if a small percentage (3.1%) reported using e-cigs also indoors. More than 72% of e-cig/HTP users have never been advised by a doctor to quit using these products, and only a minority (<20%) were planning to quit using them in the following six months.

Considering both ever users of e-cigs (1107 students) and HTPs (609 students), most started using electronic devices as an alternative to traditional cigarettes (43 and 46%, respectively), because they considered these products less dangerous for health than traditional cigarettes (34 and 44%), as an aid to quit traditional cigarettes (31 and 30%), or because these products are trendy (35 and 29%). However, 31.9% of e-cig and 27.2% of HTP users started or went back to smoking traditional cigarettes after starting to use electronic devices. Notably, 21% of e-cig and 12.5% of HTP users did not smoke traditional cigarettes before using electronic devices.

### 3.5. Passive Smoking

Passive smoke exposure was investigated among all students, irrespective of their active smoking status, by the question “Over the last week, have you been exposed to passive smoke continuously for at least 10 min?”. As much as 41% of students reported having been exposed in the previous week, with differences among faculties (*p* < 0.001) (Table 4). In particular, PESS students reported exposure more frequently than students from Hum, Med, Pha, and STE. Most students (67%) were exposed only outdoors, but a relatively high percentage (27%) also indoors, including at home (54%), in the car (32%), or in other places (i.e., public places or friend’s houses) (67%) (Supplementary Table S1E). About one-third of the students reported living with smokers, and two-thirds spent leisure time with smokers on a regular base. Again, differences were noted among faculties (*p* < 0.001), with students from PESS or Law more frequently reporting both living and spending leisure time with smokers than other faculties.

A total smoking ban was present in many students’ houses (42.4%) both for tobacco cigarettes and e-cigs/HTPs, with differences among faculties (*p* < 0.001) (Table 4). For traditional cigarettes, the prevalence was higher among Med than Law, PESS, Hum, STE, and LMIC students, while for e-cigs/HTPs, the prevalence was higher again among Med than Law, PESS, Hum, and LMIC students, and among Sports than PESS and LMIC students. It was allowed to smoke tobacco cigarettes without restrictions in 5.6% of houses, while for e-cigs and HTPs the percentage was higher (15%). For e-cigs/HTPs, the prevalence was higher among students from PESS and Hum than Med.

### 3.6. Awareness of Smoking Health-Related Issues and Role of Healthcare Professionals

Almost all students (99.2%) considered active smoking of traditional cigarettes dangerous for health (Supplementary Table S2). As regards e-cigs and HTPs, the great majority considered active smoking dangerous for health (74.5 and 81.6%, for e-cigs and HTPs, respectively), and only a minority considered smoking e-cigs or HTPs dangerous only in particular health conditions (e.g., pregnancy or illness) (2.2 and 1.3%), or no danger at all (1.5 and 0.4%). Notably, a relatively high percentage of students did not have an opinion on this topic (21.8% and 16.6%). Differences were found among faculties (*p* < 0.001): for e-cig the positive responses were higher in students from Med than from PESS, Hum, STE, Agr, Vet, and LMIC. Moreover, the prevalence of student without an opinion on this regard was higher in students from Hum, STE, Agr, and Vet than Med. Similar results were found for HTPs.

As regards passive smoking, the great majority (94%) considered passive smoking of traditional cigarettes dangerous for health (Supplementary Table S3), with a higher prevalence among students from Med, than Law, PESS, Hum, STE, and Agr. Moreover, a higher percentage of students from Law, PESS, and Hum than from Med or Pha considered passive smoking dangerous only in particular health situations. Only a very low percentage of students considered passive smoking not dangerous, or they did not have an opinion.

For e-cigs and HTPs, 55.4% of students considered passive smoking dangerous for health, with a higher prevalence among students from Med, than PESS, Hum, STE, Agr, and LMIC. Notably, as well as 28% did not have an opinion in this regard (Supplementary Table S3). A lower percentage of students considered passive smoking from e-cigs or HTPs dangerous only in particular situations (6.9%) or not dangerous at all (9.3%).

The questionnaire also investigated the role of healthcare professionals (Supplementary Table S4): 55.4% (range 48% for PESS to 75% for Medicine, *p* < 0.001) of students believed that healthcare professionals serve as role models for their patients and society regarding smoking habits and they should regularly advise their smoking patients to stop smoking tobacco cigarettes (85.6%, range 81% for Hum to 93% for Medicine and Sports, *p* < 0.001) or e-cigs/HTPs (70.8%, range 66% for Hum to 81% for Medicine, *p* < 0.001).

Finally, only students from Med had access to a question investigating their specific educational needs on smoking issues: 75% reported they would like more information on the health effects of new tobacco products, 67% on smoking cessation techniques, 55% on the damage induced by passive smoking and 40% on the health effects caused by traditional cigarette smoking. Only 8% reported that their training on these topics was satisfactory (Supplementary Table S1F).

### 3.7. Knowledge and Attitudes towards Smoking Legislation and Policy

The majority of students (96%) reported that the Italian regulatory provision banning smoking indoors in public places is useful for protecting the health of nonsmokers, 79% knew that smoking is prohibited in cars in the presence of children and pregnant women, 85% knew that it is forbidden to sell e-cigarettes with nicotine to minors under 18 years of age, 89% were aware of the ban on throwing cigarette butts on the ground, and 91% (range 83% for Sport to 94% for Hum, *p* < 0.001) of the damage caused by cigarette butts dispersed in the environment (Table 5 and Table S1G). A minority (18%) thought that shock images on cigarette packs are effective as a health warning.

Less than half (48%, range 40% for Vet to 52% for Law, *p* = 0.010) were aware of the smoking ban of all smoking products in the outdoor areas of schools and universities, and only 49% (range 32% for Sport to 54% for Med, *p* < 0.001) knew that the University of Milan has an internal regulation about it. Only a minority of students (3%) reported that the ban on smoking cigarettes and e-cigs/HTPs in outdoor areas of the University is complied with, and about 20% of students did not have an opinion in this regard.

Finally, 64% (range 56% for Sport to 70% for Pha, *p* < 0.001) of students would like greater control over compliance with existing bans at University, 54% (range 47% for Law to 64% for Med, *p* < 0.001) welcomed cessation aid for smokers, 40% informative campaigns on the harm of smoking, and 25% (range 20% for STE to 33% for Med, *p* < 0.001) specific courses on smoking issues. 

## 4. Discussion

In this paper, we investigated smoking habits, attitudes, and knowledge among students from the University of Milan as the first initiative of the project “La Statale Smoke-free”.

To the best of our knowledge, this is the first study in Italy involving the entire student population of a university and investigating several aspects related to smoking. Previous studies in Italy had been limited to single university courses, and almost exclusively in Medicine and Health professions [19,20,21,22,23]. Even if the response rate was quite moderate (11%), the study participants were broadly representative of the student population for sex, age, and faculty of enrollment (Table 1). This is of relevance, as the information collected in this study will be useful to propose anti-smoking actions aimed at the entire student community. The participation rate in this study was higher than that obtained in similar studies based on web surveys, with a reported response rate in the 3–8% range for studies conducted in the US [24], New Zealand [25], Poland [26], and Bosnia Herzegovina [27]. This result can be partly due to the invitation letter, sent by the Rector, which prompted the students to participate. On the other hand, in our pilot study, a much higher response rate (78%) was obtained [17], probably because the survey involved the relatively small degree course of Obstetrics, whose students were particularly interested in the topic and were warmly invited by the course chair to participate. As we observed in the pilot study, the four reminders to the first call contributed to boosting the participation rate, passing from 4.0% at the first call to 11% after the fourth call. Higher response rates (up to 90%) were obtained in studies involving a low number of students [22,28,29] using other engagement actions, including printed questionnaires administered during classes or lectures, however, this strategy was not applicable in the present survey.

Overall, the percentage of exclusively tobacco smokers (19.3%) (Table 2) was slightly lower than among young Italians aged 15–24 years (21.1%) and among the Italian adult population (>15 years old) (24.2%) [30]. Moreover, most smokers (77%) smoked less than 10 cigarettes/day in comparison with a mean of 11.5 in the general population and had a mild nicotine addiction at the Fagerström test. Altogether, these observations could be indicative of a higher awareness of health issues generally observed in higher educated individuals. However, the prevalence of smokers here found was almost double or higher than among university students in the US (7.8%) [24], Eastern European countries (12.3%) [29], New Zealand (10.4%) and Australia (8.9%) [31], Oman (11.3%) [32], Jordan (11.3%) [33], and Poland (10.3%) [26], but similar to Germany and Hungary (18%) [28] and much lower than in Bosnia Herzegovina (38.8%) [27].

The percentage of smokers was different among faculties, and it was higher among students from the PESS, Humanities, and Law faculty than in all the other faculties. Differences among faculties have been reported very seldom: differently from our study, Šljivo et al. reported a lower prevalence of smokers in the Social Faculty (17%) than in the Technical (23.4%) and Medicine faculty (60%) in Bosnia Herzegovina [27], while Brożek et al. reported no differences between medical and non-medical students (12%) in Eastern European countries [29]. As regards the Medicine faculty, the percentage of smokers here found was much lower than in previous studies conducted in Italy among medical students, where the percentage of smokers in the 25–45% range had been reported [19,20,21,22,23,34,35].

While there was no difference among faculties as regards the smoking intensity or the quitting tentative, the students from Med reported being advised by a doctor to quit smoking more frequently than other students (Table 3). As medical students have hospital training periods as part of their classes, this result could be a consequence of their frequent contact with doctors. However, it appears that getting more advice did not translate into more students willing to quit, as the percentage of students planning to quit was similar among faculties.

As regards e-cigs or HTPs, the percentage of current users (about 4%), was slightly higher than national data (2.4% and 3.3% for e-cigs and HTPs, respectively), in agreement with the use of these products mostly by young people [4,5]. It is interesting to note that, while more than 40% of ever users declared to start using these products as an alternative to traditional cigarettes and 30% to quit traditional smoking, almost 30% started or resumed smoking traditional cigarettes after starting to use electronic products. These data support previous observations that these products represent a gateway for nicotine addiction in never users [5,36]. Moreover, 30% of students declared to use these electronic devices because they are trendy, confirming that the aggressive market campaigns of producers, aimed at presenting these products as unique, with sleek designs and causing less harm than traditional cigarettes, are successful in gaining new users among young people. Differences among faculties were observed for these products too, with a higher percentage of ever users among Law and PESS students than Med and other scientific faculties. Moreover, while most students were aware of health issues related to active smoking of traditional cigarettes, the highest prevalence of students aware of risks from e-cigs and HTPs was found among Med students (Table S2). This result, together with the lower percentage of students from Med reporting no opinion about these issues, shows the need for more education and raising awareness in those faculties that do not deal with health issues.

Exposure to passive smoke is a relevant health issue, as it has been associated with several illnesses and has been classified as carcinogenic to humans [37]. We found that exposure to passive smoke is a common experience for students, as a high percentage reported being exposed to passive smoke, living with smokers, and spending leisure time with smokers (Table 4). Again, students from PESS or Law reported recent exposure, living, and spending leisure time with smokers more frequently than other students. The high percentage of students reporting awareness about health issues related to passive smoke (mean 94%, Table S3) shows that informative campaigns and/or school education have been effective in spreading general knowledge, but this was not completely sufficient to make students adopt different behaviors. This suggests that university policy should be aimed not only at disseminating information about passive smoke dangers, but also at promoting smoke-free policies and adopting effective interventions to address and possibly decrease passive exposure.

The same level of awareness was not found for e-cigs/HTPs, as only 55% considered passive exposure to vaping bad for health. Although recognized toxicants and carcinogens have been found in e-cig/HTP emissions, even in concentrations lower than those found in emissions from traditional cigarettes [38], these products are perceived as less harmful than cigarettes and they are often marketed with this claim. The limited data on the human health effects and the lack of long-term studies on chronic diseases [39,40] contribute to increasing the uncertainty about these products and their diffusion among young adults. The lack of awareness may be the basis of a wider acceptance of e-cigs/HTPs among students, as shown by the higher percentage accepting vaping rather than smoking everywhere at home (15% vs. 5.6%). Notably, students from Med, although they were the most aware of these issues, required more information on the health effects of new tobacco products as their primary educational need.

In comparison with the general population, students reported a lower percentage of houses with a total smoking ban for traditional cigarettes than in homes with children in Milan (42% vs. 63%) [41]: as students often live alone or with other students, this reflects the fact that rules at their home are less strict than in homes with children. Students from Med were the most aware of passive smoking-related harms both for traditional cigarettes and for e-cigs/HTPs, and they were also the students with the lowest acceptance of smoking or vaping in their homes. Based on this result, we could speculate that a deeper knowledge leads to more responsible behaviors.

An interesting result of this survey is that the percentage of students considering healthcare professionals as role models for their patients and society regarding smoking habits was significantly higher among students from the Med faculty than in the other faculties (Table S4). This result suggests that Med students are aware of their future role, both as professionals that can give appropriate advice in promoting a healthy lifestyle and as institutional figures that can influence patients with their behaviors, and they feel acknowledged in their role by society, however the same is not true for students from the other faculties, except for Pha students. It is likely that considerations about “personal freedom” or “perceived rights of smokers to smoke” largely influence these opinions, as observed in students opposing smoking bans in colleges [42,43]. The higher percentage of Med students than other students reporting that healthcare professionals should regularly advise to quit smoking or vaping and the reported educational need on smoking cessation techniques seem to confirm this observation. It is striking to note that the percentage of opponents to healthcare professionals advising patients to stop smoking or vaping was the highest among those faculties with the highest percentage of smokers, PESS, and Law.

Finally, while most students were aware of the national legislation in force and of its motivation, only about half of them were aware of regulations regarding schools and universities (Table 5). This finding partly explains the observed lack of compliance with smoking and tobacco ban in outdoor areas of the university and underlines the need for better communication of anti-smoking policies. Given these results, one of the first actions of the project “La Statale Smoke-free” will be the launch of a communication campaign to raise awareness of these topics. Students reported being interested in any initiatives that the University of Milan could promote to help smokers quit smoking and to protect the health of non-smokers. Once more, the request for initiatives was higher among students from Med, emphasizing their trust in the effectiveness of prevention in combating the exposure to tobacco smoke.

This study is the first to examine the prevalence, attitudes, and patterns of smoking, both active and passive, in the entire student population of a big university in Italy. Moreover, differences among faculties were highlighted for the first time. The main limitation of the study is that students participated on a volunteer base, so it was not possible to control participation for some socioeconomics or personal characteristics that could influence smoking habits. However, the survey involved a relatively large sample of students whose characteristics, in terms of age, sex, and faculty of enrollment were closely similar to the students at the University of Milan, so the conclusions are likely to be generalizable to the student population of this University. The main strength of the study is that the survey was conducted using a web questionnaire previously validated and pilot tested and included an extended panel of questions that covered different areas of interest [17].

## 5. Conclusions

In conclusion, this study highlighted different prevalence and opinions about both active and passive smoking of various tobacco products in students from different faculties of the same University. Students from the Law and PESS faculty were more frequently smokers or exposed to passive smoke than students from the Med faculty. The last ones, on the contrary, were the most conscious of harms from both active and passive smoking and vaping. This finding could be the consequence of the evidence that students from faculties different from Med receive, during their academic curricula, less (or no) information about smoking and health-related issues and/or that they are less interested in health issues anyway, while medical students give great importance to health, as expected, both for their specific education and personal inclination. Different course organizations (i.e., no compulsory classes, little or no training periods, or laboratory classes) could also partially explain the above-reported observations. The results of this study suggest that prevention campaigns addressed to students should consider the specific study curricula of the different faculties and give information tailored to the different educational needs to efficiently support students in their healthy choice.

## Figures and Tables

**Table 1:** Comparison between the study participants (N = 6605) and the student population at the University of Milan (N = 64,801) as regards sex, age, and faculty of enrollment, and participation rate.

Characteristics		Students Participating to the Survey N (%)	Student Population N (%)	Participation Rate (%)
**Sex**	**Male**	2408 (36.5)	26,233 (40.5)	9.2
	**Female**	4105 (62.1)	38,568 (59.5)	10.6
	**intersex**	19 (0.3%)	n.a.	-
	**not declared**	73 (1.1%)	n.a.	-
**Age (year)**	**18–25 years**	5555 (84.1)	51,128 (78.9)	10.9
	**≥26 years**	1050 (15.9)	13,673 (21.1)	7.7
**Faculty**	**Law**	441 (6.7)	6835 (10.5)	6.5
	**PESS**	891 (13.5)	8240 (12.7)	10.8
	**Hum**	1447 (21.9)	15,795 (24.4)	9.2
	**Med**	969 (14.7)	8132 (12.5)	11.9
	**Pha**	486 (7.4)	3946 (6.1)	12.3
	**STE**	1353 (20.5)	9978 (15.4)	13.6
	**Agr**	350 (5.3)	3485 (5.4)	10.0
	**Vet**	170 (2.6)	1492 (2.3)	11.4
	**LMIC**	401 (6.1)	5578 (8.6)	7.2
	**Sport**	97 (1.5)	1320 (2.1)	7.3

**Table 2:** Smoking habits among all study participants (N = 6605) and students stratified by sex, age, and faculty.

Characteristics		Nonsmokers			Smokers/Vapers			
		Never Smokers N (%)	Former Smokers N (%)	Total Nonsmokers ^1^ N (%)	Tobacco Cigarettes Only N (%)	e-cigs/HTPs Only N (%)	Dual ^2^ N (%)	Total Smokers/Vapers ^3^ N (%)
**All subjects**		4212 (63.8)	687 (10.4)	4899 (74.2)	1278 (19.3)	184 (2.8%)	244 (3.7%)	1706 (25.8)
**Sex**	**Male**	1430 (59.4)	275 (11.4)	1705 (70.8)	538 (22.3)	70 (2.9)	95 (3.9)	703 (29.2)
	**Female**	2734 (66.6)	403 (9.8)	3137 (76.4)	710 (17.3)	114 (2.8)	144 (3.5)	968 (23.6)
**Age (years)**	**18–25**	3608 (65.0)	485 (8.7)	4093 (73.7)	1097 (19.7)	153 (2.8)	212 (3.8)	1462 (26.3)
	**≥26**	604 (57.5)	202 (19.3)	806 (76.7)	181 (17.2)	31 (3.0)	32 (3.0)	244 (23.3)
**Faculty**	**Law**	232 (52.6)	61 (13.8%)	293 (66.4)	103 (23.4) ^D,E,F^	18 (4.1)	27 (6.1)	148 (33.6) ^D,E,F^
	**PESS**	481 (54.0)	97 (10.9)	587 (64.9)	235 (26.4) ^D,E,F,G,H^	35 (3.9)	43 (4.8)	313 (35.1) ^D,E,F,G,H,I^
	**Hum**	841 (58.1)	169 (11.7)	1010 (69.8)	354 (24.5) ^D,E,F,H^	31 (2.1)	52 (3.6)	437 (30.2) ^D,E,F^
	**Med**	692 (71.4) ^A,B,C^	93 (9.6)	785 (81.0)	144 (14.9)	19 (2.0)	21 (2.2)	184 (19.1)
	**Pha**	339 (69.8) ^A,B,C^	49 (10.1)	388 (79.9)	57 (11.7)	19 (3.9)	22 (4.5)	98 (20.1)
	**STE**	942 (69.6) ^A,B,C^	120 (8.9)	1062 (78.5)	215 (15.9)	34 (2.5)	42 (3.1)	291 (21.5)
	**Agr**	232 (66.3) ^A,B^	40 (11.4)	272 (77.7)	60 (17.1)	8 (2.3)	10 (2.9)	78 (22.3)
	**Vet**	119 (70.0) ^A,B^	14 (8.2)	133 (78.2)	22 (12.9)	6 (3.5)	9 (5.3)	37 (21.7)
	**LMIC**	264 (65.8) ^A,B^	37 (9.2)	301 (75.0)	74 (18.5)	14 (3.5)	12 (3.0)	100 (25.0)
	**Sport**	70 (72.2) ^A,B^	7 (7.2)	77 (79.4)	14 (14.4)	0	6 (6.2)	20 (20.6)

^1^ = sum of never smokers and former smokers; ^2^ = smokers and e-cig/HTP users; ^3^ = sum of smokers, exclusively vapers, and dual users; ^A^ = higher than Law, ^B^ = higher than PESS, ^C^ = higher than Hum, ^D^ = higher than Med, ^E^ = higher than Pha, ^F^ = higher than STE, ^G^ = higher than Agr, ^H^ = higher than Vet, ^I^ = higher than LMIC (pairwise comparisons between faculties, only differences with *p* < 0.05 are marked with capital letters).

**Table 3:** Active traditional cigarette smoking, including exclusively cigarette smokers and dual smokers (1517 responders).

	How Often Do You Currently Smoke?	Have You Ever Tried to Quit Traditional Cigarettes?	Have You Ever Been Advised by a Doctor or Other Healthcare Professional to Quit Smoking Traditional Cigarettes?			Are You Planning to Quit Smoking Traditional Cigarettes in the Next Six Months?		
	Daily ^a^ N (%)	Yes ^b^ N (%)	Yes N (%)	No N (%)	Don’t Remember N (%)	Yes N (%)	No N (%)	Don’t Know N (%)
**All subjects**	1173 (77.3)	899 (59.4)	554 (36.6)	812 (53.7)	146 (9.7%)	322 (21.3)	610 (40.3)	580 (38.4)
**Law**	102 (78,5)	78 (60.9)	50 (39.1)	58 (45.3)	20 (15.6)	22 (17.2)	55 (43.0)	51 (39.8)
**PESS**	217 (78.6)	163 (59.3)	86 (31.4)	162 (59.1) ^D^	26 (9.5)	75 (27.4)	108 (39.4)	91 (33.2)
**Hum**	316 (78.0)	228 (56.3)	149 (36.8)	211 (52.1)	45 (11.1)	69 (17.0)	174 (43.0)	162 40.0)
**Med**	123 (75.0)	102 (62.2)	87 (53.0) ^B,C,E,F^	66 (40.2)	11 (6.7)	38 (23.2)	62 (37.8)	64 (39.0)
**Pha**	58 (73.4)	46 (59.0)	22 (28.6)	47 (61.0)	8 (10.4)	21 (27.3)	26 (33.8)	30 (39.0)
**STE**	207 (80.5)	157 (61.1)	81 (31.5)	159 (61.9) ^D^	17 (6.6)	53 (20.6)	106 (41.2)	98 (38.1)
**Agr**	55 (78.6)	42 (60.0)	29 (41.4)	33 (47.1)	8 (11.49	11 (15.7)	33 (47.1)	26 (37.1)
**Vet**	22 (71.0)	19 (61.3)	8 (25.8)	20 (64.5)	3 (9.7)	4 (12.9)	11 (35.5)	16 (51.6)
**LMIC**	60 (69.8)	48 (55.8)	33 (38.4)	47 (54.7)	6 (7.0)	21 (24.4)	26 (30.2)	39 (45.3)
**Sport**	13 (65.0)	16 (80.0)	9 (45.0)	9 (45.0)	2 (10.0)	8 (40.0)	9 (45.0)	3 (15.0)
*p* *	0.486	0.677	0.001			0.041		

^a^ = vs. non-daily; ^b^ = vs. no; * = *p* values represent the significance of chi-square tests for comparison among faculties; ^B^ = higher than PESS, ^C^ = higher than Hum, ^D^ = higher than Med, ^E^ = higher than Pha, ^F^ = higher than STE (pairwise comparisons between faculties, only differences with *p* < 0.05 are marked with capital letters).

**Table 4:** Passive smoke exposure and smoking ban at home (6579 responders).

	Have You Been Exposed for at Least 10 Min over the Last Week?	Do You Live with Smokers?	Do You Usually Spend Leisure Time with Smokers?	In Your House, Traditional Cigarettes Are:				In Your House, e-cigs/HTPs Are:			
	Yes ^a^ N (%)	Yes ^a^ N (%)	Yes ^a^ N (%)	Totally Banned N (%)	Allowed in Some Rooms N (%)	Allowed Only Outdoors N (%)	Allowed Everywhere N (%)	Totally Banned N (%)	Allowed in Some Rooms N (%)	Allowed Only Outdoors N (%)	Allowed Everywhere N (%)
**All subjects**	2698 (41.0)	2269 (34.4)	4358 (66.3)	2786 (42.4)	514 (7.8)	2907 (44.2)	367 (5.6)	2785 (42.4)	611 (9.3)	2195 (33.4)	983 (15.0)
**Law**	193 (44.3)	172 (39.4) ^D^	318 (72.9) ^D,E,F^	165 (37.8)	44 (10.1) ^D^	209 (47.9)	18 (4.1)	165 (37.8)	57 (13.1) ^D^	140 (32.1)	74 (17.0)
**PESS**	430 (48.6) ^C.D.E.F^	329 (37.2) ^D^	662 (74.9) ^C,D,E,F,G,H,I^	344 (38.9)	79 (8.9)^D^	405 (45.8)	56 (6.3)	349 (39.5)	105 (11.9) ^D^	279 (31.6)	151 (17.1) ^D^
**Hum**	561 (38.9)	541 (37.5) ^D,F^	974 (67.5)	527 (36.6)	145 (10.1) ^D;H^	676 (46.9) ^D^	93 (6.5)	548 (38.0)	148 (10.3) ^D^	504 (35.0)	241 (16.7) ^D^
**Med**	383 (39.6)	284 (29.4)	601 (62.2)	491 (50.8) ^A,B,C,F,I^	46 (4.8)	388 (40.2)	41 (4.2)	480 (49.7) ^A,B,C,I^	46 (4.8)	329 (34.1)	111 (11.5)
**Pha**	184 (38.1)	164 (33.9)	302 (62.5)	229 (47.4) ^C^	35 (7.2)	196 (40.6)	23 (4.8)	218 (45.1)	45 (9.3) ^D^	151 (31.3)	69 (14.3)
**STE**	511 (37.8)	427 (31.6)	849 (62.8)	593 (43.9) ^C^	93 (6.9)	584 (43.3)	80 (5.9	586 (43.4)	119 (8.8) ^D^	457 (33.9)	188 (13.9)
**Agr**	153 (43.8)	109 (31.1)	219 (62.8)	164 (47.1) ^C^	30 (8.6)	137 (39.4)	17 (4.9)	159 (45.7)	28 (8.0)	105 (30.2)	56 (16.1)
**Vet**	61 (35.9)	61 (35.9)	106 (62.4)	73 (42.9)	4 (2.4)	86 (50.6)	7 (4.1)	77 (45.3)	11 (6.5)	64 (37.6)	18 (10.6)
**LMIC**	176 (44.1)	152 (38.1)	263 (65.9)	148 (37.1)	36 (9.0)	189 (47.4)	26 (6.5)	149 (37.3)	47 (11.8) ^D^	143 (35.8)	60 (15.0)
**Sport**	46 (47.4)	30 (30.9)	64 (66.0)	52 (53.6) ^C^	2 (2.1)	37 (38.1)	6 (6.2)	54 (55.7) ^C,I^	5 (5.2)	23 (23.7)	15 (15.5)
*p* *	<0.001	<0.001	<0.001	<0.001				<0.001			

^a^ = vs. no; * = *p* values represent the significance of chi-square tests for comparison among faculties; ^A^ = higher than Law, ^B^ = higher than PESS, ^C^ = higher than Hum, ^D^ = higher than Med, ^E^ = higher than Pha, ^F^ = higher than STE, ^G^ = higher than Agr, ^H^ = higher than Vet, ^I^ = higher than LMIC (pairwise comparisons between faculties, only differences with *p* < 0.05 are marked with capital letters).

**Table 5:** Knowledge and attitudes towards Italian smoking legislation, and educational needs (6544 responders).

	Are You Aware That It Is Forbidden to Throw Cigarette Butts on the Ground?	Are You Aware of the Damage Caused by Cigarette Butts in the Environment?	Are You Aware That It Is Forbidden to Smoke and Vape in the Outdoor Areas of Schools and Universities?	Are You Aware That the University of Milan Has Anti-Smoking Regulations?	What Initiatives Could the University of Milan Undertake to Help Smokers Quit Smoking and Protect the Health of Non-Smokers?			
	Yes ^a^ N (%)	Yes ^a^ N (%)	Yes ^a^ N (%)	Yes ^a^ N (%)	Informative Campaigns	Greater Control over Compliance with Bans	Courses on Smoking Issues	Cessation Aid
**All subjects**	5789 (88.5)	5977 (91.3)	3117 (47.6)	3233 (49.4)	2064 (39.8)	4166 (63.7)	1627 (24.9)	3560 (54.4)
**Law**	397 (91.1)	408 (93.6) ^J^	226 (51.8)	233 (53.4) ^J^	170 (38.5)	280 (63.5)	113 (25.6)	207 (46.9)
**PESS**	771 (87.3)	797 (90.3)	400 (45.3)	391 (44.3)	355 (39.8)	534 (59.9)	191 (21.4)	468 (52.5)
**Hum**	1288 (89.8)	1344 (93.7) ^D,J^	703 (49.0)	727 (50.7) ^J^	566 (39.1)	863 (59.6)	368 (25.4)	743 (51.3)
**Med**	843 (88.2)	847 (88.6)	485 (50.7)	520 (54.4) ^B,I,J^	399 (41.2)	654 (67.5) ^B,C^	319 (32.9) ^B,C,F,G,I^	617 (63.7) ^A,B,C,E,F^
**Pha**	419 (87.1)	439 (91.3)	246 (51.1)	245 (50.9) ^J^	209 (43.0)	338 (69.5) ^B,C^	128 (26.3)	245 (50.4)
**STE**	1182 (87.8)	1218 (90.4)	613 (45.5)	668 (49.6) ^J^	515 (38.1)	843 (62.3)	275 (20.3)	707 (52.3)
**Agr**	305 (88.7)	318 (92.7)	148 (43.0)	159 (46.2)	130 (37.1)	212 (60.6)	75 (21.4)	196 (56.0)
**Vet**	151 (89.3)	157 (92.9)	68 (40.2)	82 (48.5)	63 (37.1)	118 (69.4)	42 (24.7)	101 (59.4)
**LMIC**	345 (86.7)	369 (92.7)	184 (46.2)	177 (44.5)	151 (37.7)	270 (67.3)	89 (22.2)	220 (54.9)
**Sport**	88 (91.7)	80 (83.3)	44 (45.8)	31 (32.3)	46 (47.4)	54 (55.7)	27 (27.8)	56 (57.7)
*p* *	0.308	<0.001	0.010	<0.001	0.397	<0.001	<0.001	<0.001

^a^ = vs. no; * = *p* values represent the significance of chi-square tests for comparison among faculties; ^A^ = higher than Law, ^B^ = higher than PESS, ^C^ = higher than Hum, ^D^ = higher than Med, ^E^ = higher than Pha, ^F^ = higher than STE, ^G^ = higher than Agr, ^I^ = higher than LMIC, ^J^ = higher than Sport (pairwise comparisons between faculties, only differences with *p* < 0.05 are marked with capital letters).

## Data Availability

The data presented in this study are available in Supplementary Materials.

## Supplementary Materials

The following are available online at https://www.mdpi.com/article/10.3390/ijerph191912527/s1. Table S1B: Answers from Section B of the questionnaire: Active smoking of traditional tobacco cigarettes; Table S1C: Answers from Section C of the questionnaire: Former smoker of traditional tobacco cigarettes; Table S1D: Answers from Section D of the questionnaire: Electronic cigarettes or HTP users; Table S1E: Answers from Section E of the questionnaire: Passive smoking; Table S1F: Answers from Section F of the questionnaire: Knowledge of smoking health-related issues and role of healthcare professionals; Table S1G: Answers from Section G of the questionnaire: Knowledge and attitudes towards Italian smoking legislation, and educational needs; Table S2: Awareness of health issues related to active smoking; Table S3: Awareness of health issues related to passive smoking; Table S4: Role of healthcare professionals.

## References

[1] (2019). Global Burden of Disease (GBD). https://www.thelancet.com/gbd/summaries.

[2] Ministero Della Salute (2019). Prevenzione e Controllo Del Tabagismo-Rapporto Anno 2018 [In Italian]. https://www.salute.gov.it/portale/documentazione/p6_2_2_1.jsp?lingua=italiano&id=2851.

[3] Gallus S., Muttarak R., Martínez-Sánchez J.M., Zuccaro P., Colombo P., La Vecchia C. (2011). Smoking prevalence and smoking attributable mortality in Italy, 2010. Prev. Med..

[4] Boakye E., Osuji N., Erhabor J., Obisesan O., Osei A.D., Mirbolouk M., Stokes A.C., Dzaye O., El Shahawy O., Hirsch G.A. (2022). Assessment of Patterns in e-Cigarette Use among Adults in the US, 2017–2020. JAMA Netw. Open.

[5] Liu X., Lugo A., Davoli E., Gorini G., Pacifici R., Fernández E., Gallus S. (2019). Electronic cigarettes in Italy: A tool for harm reduction or a gateway to smoking tobacco?. Tob. Control.

[6] McMillen R., Maduka J., Winickoff J. (2012). Use of Emerging Tobacco Products in the United States. J. Environ. Public Health.

[7] World Health Organization (WHO) (2003). WHO Framework Convention on Tobacco Control (WHO FCTC). https://fctc.who.int/who-fctc/overview.

[8] Italian Legislation, Law Decree 104, Art. 4, 12 September 2013 (Misure Urgenti in Materia di Istruzione, Universita’ e Ricerca. (13G00147)) Gazzetta Ufficiale Serie Generale n.214, 12 September 2013, Enforced from 12 September 2013. https://www.gazzettaufficiale.it/eli/id/2013/09/12/13G00147/sg.

[9] ESPAD Group (2019). ESPAD Report 2019: Results from the European School Survey Project on Alcohol and Other Drugs.

[10] Ling P.M., Glantz S.A. (2002). Why and How the Tobacco Industry Sells Cigarettes to Young Adults: Evidence From Industry Documents. Am. J. Public Health.

[11] Bennett B.L., Deiner M., Pokhrel P. (2017). College anti-smoking policies and student smoking behavior: A review of the literature. Tob. Induc. Dis..

[12] Jenssen B.P., Walley S.C., Groner J.A., Rahmandar M., Boykan R., Mih B., Marbin J.N., Caldwell A.L. (2019). Section on Tobacco Control. E-Cigarettes and Similar Devices. Pediatrics.

[13] Meier E., Lechner W.V., Miller M.B., Wiener J.L. (2013). Changes in Smokeless Tobacco Use over Four Years Following a Campus-Wide Anti-tobacco Intervention. Nicotine Tob. Res..

[14] Seo D.-C., Macy J.T., Torabi M.R., Middlestadt S.E. (2011). The effect of a smoke-free campus policy on college students’ smoking behaviors and attitudes. Prev. Med..

[15] Lee J.G., Ranney L.M., Goldstein A.O. (2013). Cigarette butts near building entrances: What is the impact of smoke-free college campus policies?. Tob. Control.

[16] Burns S., Hart E., Jancey J., Hallett J., Crawford G., Portsmouth L. (2016). A cross sectional evaluation of a total smoking ban at a large Australian university. BMC Res. Notes.

[17] Campo L., Vecera F., Fustinoni S. (2021). Validation of a Questionnaire to Assess Smoking Habits, Attitudes, Knowledge, and Needs among University Students: A Pilot Study among Obstetrics Students. Int. J. Environ. Res. Public Health.

[18] (2006). The GTSS Collaborative Group Tobacco use and cessation counselling: Global Health Professionals Survey Pilot Study, 10 countries, 2005. Tob. Control.

[19] Rodakowska E., Mazur M., Baginska J., Sierpinska T., La Torre G., Ottolenghi L., D’Egidio V., Guerra F. (2020). Smoking Prevalence, Attitudes and Behavior among Dental Students in Poland and Italy. Int. J. Environ. Res. Public Health.

[20] Prigitano A., Binda S., Pariani E. (2020). Tobacco and e-cigarette smoking habits among Italian healthcare students. Ann. Ig.

[21] Armstrong G.W., Veronese G., George P.F., Montroni I., Ugolini G. (2017). Assessment of Tobacco Habits, Attitudes, and Education Among Medical Students in the United States and Italy: A Cross-sectional Survey. J. Prev. Med. Public Health.

[22] La Torre G., Kirch W., Bes-Rastrollo M., Ramos R., Czaplicki M., Gualano M., Thümmler K., Ricciardi W., Boccia A. (2011). Tobacco use among medical students in Europe: Results of a multicentre study using the Global Health Professions Student Survey. Public Health.

[23] Garzillo E.M., Monaco M.G.L., Corvino A.R., Giardiello A., Arnese A., Napolitano F., Di Giuseppe G., Lamberti M. (2022). Smoking Habits and Workplace Health Promotion among University Students in Southern Italy: A Cross-Sectional Pilot Investigation. Int. J. Environ. Res. Public Health.

[24] Latimer L.A., Batanova M., Loukas A. (2013). Prevalence and Harm Perceptions of Various Tobacco Products among College Students. Nicotine Tob. Res..

[25] Wamamili B., Wallace-Bell M., Richardson A., Grace R.C., Coope P. (2019). Cigarette smoking among university students aged 18–24 years in New Zealand: Results of the first (baseline) of two national surveys. BMJ Open.

[26] Pazdro-Zastawny K., Dorobisz K., Bobak-Sarnowska E., Zatoński T. (2022). Prevalence and Associated Factors of Cigarette Smoking Among Medical Students in Wroclaw, Poland. Risk Manag. Health Policy.

[27] Šljivo A., Ćetković A., Hašimbegović-Spahić D., Mlačo N., Mujičić E., Selimović A. (2022). Patterns of cigarette, hookah and other tobacco product consumption habits among undergraduate students of the University of Sarajevo before the COVID-19 outbreak in Bosnia and Hercegovina, a cross-sectional study. Ann. Ig.

[28] Balogh E., Faubl N., Riemenschneider H., Balázs P., Bergmann A., Cseh K., Horváth F., Schelling J., Terebessy A., Wagner Z. (2018). Cigarette, waterpipe and e-cigarette use among an international sample of medical students. Cross-sectional multicenter study in Germany and Hungary. BMC Public Health.

[29] Brożek G.M., Jankowski M., Lawson J.A., Shpakou A., Poznański M., Zielonka T.M., Klimatckaia L., Loginovich Y., Rachel M., Gereová J. (2019). The Prevalence of Cigarette and E-cigarette Smoking among Students in Central and Eastern Europe—Results of the YUPESS Study. Int. J. Environ. Res. Public Health.

[30] Istituto Superiore di Sanità (ISS). https://www.iss.it/web/guest//comunicati-stampa//asset_publisher/fjTKmjJgSgdK/content/id/7146126.

[31] Wamamili B., Lawler S., Wallace-Bell M., Gartner C., Sellars D., Grace R.C., Courtney R., Coope P. (2021). Cigarette smoking and e-cigarette use among university students in Queensland, Australia and New Zealand: Results of two cross-sectional surveys. BMJ Open.

[32] Al Omari O., Abu Sharour L., Heslop K., Wynaden D., Alkhawaldeh A., Al Qadire M., Khalaf A. (2020). Knowledge, Attitudes, Prevalence and Associated Factors of Cigarette Smoking among University Students: A Cross Sectional Study. J. Community Health.

[33] Al-Sawalha N.A., Almomani B.A., Mokhemer E., Al-Shatnawi S.F., Bdeir R. (2021). E-cigarettes use among university students in Jordan: Perception and related knowledge. PLoS ONE.

[34] Provenzano S., Santangelo O., Grigis D., Giordano D., Firenze A. (2019). Smoking behaviour among nursing students: Attitudes toward smoking cessation. J. Prev. Med. Hyg..

[35] D’Egidio V., Patrissi R., De Vivo G. (2020). Global Health Professions Student Survey among Healthcare students: A cross sectional study. Ann Ig.

[36] Bold K.W., Kong G., Camenga D.R., Simon P., Cavallo D.A., Morean M.E., Krishnan-Sarin S. (2018). Trajectories of E-Cigarette and Conventional Cigarette Use Among Youth. Pediatrics.

[37] International Agency for Research on Cancer (IARC) (2004). IARC Monographs on the Evaluation of Carcinogenic Risks to Humans. Tobacco Smoke and Involuntary Smoking.

[38] Czogala J., Goniewicz M.L., Fidelus B., Zielinska-Danch W., Travers M.J., Sobczak A. (2013). Secondhand Exposure to Vapors from Electronic Cigarettes. Nicotine Tob. Res..

[39] Callahan-Lyon P. (2014). Electronic cigarettes: Human health effects. Tob. Control.

[40] Carlsen K.C.L., Skjerven H.O., Carlsen K.-H. (2018). The toxicity of E-cigarettes and children’s respiratory health. Paediatr. Respir. Rev..

[41] Campo L., Boniardi L., Polledri E., Longhi F., Scuffi C., Fustinoni S. (2021). Smoking habit in parents and exposure to environmental tobacco smoke in elementary school children of Milan. Sci. Total Environ..

[42] Jancey J., Bowser N., Burns S., Crawford G., Portsmouth L., Smith J. (2014). No Smoking Here: Examining Reasons for Noncompliance With a Smoke-Free Policy in a Large University. Nicotine Tob. Res..

[43] Niemeier B.S., Chapp C.B., Henley W.B. (2014). Improving Tobacco-Free Advocacy on College Campuses: A Novel Strategy to Aid in the Understanding of Student Perceptions about Policy Proposals. J. Am. Coll. Health.

