# Supplementary material for: Assessing Smoking Habits, Attitudes, Knowledge, and Needs among University Students at the University of Milan, Italy
Source: Int J Environ Res Public Health. 2022 Sep 30;19(19):12527. doi: 10.3390/ijerph191912527 (PMC9566438; doi:10.3390/ijerph191912527)
Supplement: Supplementary file 1 [file ijerph-19-12527-s001.zip › ijerph-1933925-supplementary.pdf]

Supplementary materials for:

Assessing smoking habits, attitudes, knowledge, and needs among university students at the University of Milan, Italy

Laura Campo [1*], Silvia Lumia[1], Silvia Fustinoni [1,2]

[1] EPIGET - Epidemiology, Epigenetics, and Toxicology Lab, Department of Clinical Sciences and Community Health, Università degli Studi di Milano, Italy
[2] Environmental and Industrial Toxicology Unit, Fondazione IRCCS Ca' Granda Ospedale Maggiore Policlinico, Milan, Italy
* Correspondence: laura.campo@unimi.it; Tel.: 0039-02-50620116

Table S1B. Answers from Section B of the questionnaire: Active smoking of traditional tobacco cigarettes (commercial or hand-rolled) (1517 responders).

Question	Anchors	Response
1) At what age did you start smoking traditional cigarettes? age (years), mean (min-max)		16 (9-29)
2) Do you remember why you started smoking traditional cigarettes? (You can tick more than one box.) N (%)	Peer pressure / at parties / my friends smoked	811 53.3%
	Because I tried and I liked it / it gave me pleasure	815 (53.5%)
	Grown-up feeling / sense of empowerment	316 (20.8%)
	Influence from family / there were smokers in my family	150 (9.9%)
	Influence from my partner	69 (4.5%)
	Because I was feeling anxious / stressed	388 (25.5%)
	Because it made me feel confident	250 (16.4%)
	Other, specify	52 (3.4%)
3) How often do you currently smoke? N (%)	Not every day *(go to question 3a)*	345 (22.7%)
	Every day *(go to question 3b)*	1173 (77.3%)
3a) How many traditional cigarettes do you smoke per week? N (%)	1-4	133 (38.7%)
	5-9	113 (32.8%)
	10-14	65 (18.9%)
	15-19	24 (7.0%)
	≥ di 20	9 (2.6%)
3b) How many traditional cigarettes do you smoke per day? N (%)	1-4	367 (31.3%)
	5-9	441 (37.7%)
	10-14	233 (19.9%)
	15-19	94 (8.0%)
	≥ di 20	36 (3.1%)
4) Where do you usually smoke traditional cigarettes? N (%)	Only outdoors	904 (59.7%)
	Only indoors (e.g. home, car, smoking areas of public places)	11 (0.7%)
	Both indoors and outdoors	599 (39.6%)
5) Where do you smoke traditional cigarettes on campus? (You can tick more than one box.) N (%)	I do not smoke	133 (8.7%)
	Outdoors (e.g. courtyard, balconies, external stairs, porches)	1296 (85.2%)
	In smoking areas	393 (25.8%)
	Indoors (e.g. hallways, washrooms, study areas)	4 (0.3%)
6) When you are with your family, you smoke traditional cigarettes: N (%)	openly	890 (58.8%)
	secretly	624 (41.2%)
7) Have you ever tried to quit smoking traditional cigarettes? N (%)	Yes	899 (59.4%)
	No *(go to question 9)*	615 (41.2%)
8) How long have you abstained from smoking traditional cigarettes, at most? N (%)	< 7 days	141 (15.7%)
	8 days-30 days	244 (27.2%)
	> 30 days-6 months	298 (33.2%)
	> 6 months-1 year	117 (13.0%)
	> 1 year	97 (10.8%)
9) Have you ever been advised by a doctor or other healthcare professional to quit smoking traditional cigarettes? N (%)	yes	554 (36.6%)
	No	812 (53.7%)
	I do not remember	146 (9.7%)
10) Are you planning to quit smoking traditional cigarettes in the next six months? N (%)	Yes	322 (21.3%)
	No	610 (40.3%)
	I don't know	580 (38.4%)
**Fagerström test**		
1) How long after you wake up do you smoke your first cigarette? N (%)	Within 5 minutes	37 (2.4%)
	6 to 30 minutes	211 (14.0%)
	31 to 60 minutes	273 (18.1%)
	After 60 minutes	991 (65.5%)
2) Do you find it difficult to refrain from smoking in placesa where it is forbidden? N (%)	Yes	237 (15.7)
	No	1275 (84.3%)
3) Which cigarette would you hate most to give up? N (%)	First cigarette in the morning	326 (21.6%)
	All other cigarettes	1186 (78.4%)
4) How many cigarettes do you smoke per day? N (%)	10 or less	1160 (76.7%)
	11-20	317 (21.0%)
	21-30	32 (2.1%)
	31 or more	3 (0.2%)
5) Do you smoke more frequently during the first hour after waking up than during the rest of the day?	Yes	85 (5,6%)
	No	1427 (94.4%)

N (%)		
6) Do you smoke even if you are so ill that you are in bed most of the day?<br>N (%)	Yes	338 (22.4%)
	No	1174 (77.6%)

Table S1C. Answers from Section C of the questionnaire: Former smoker of traditional tobacco cigarettes (925 responders).

Question	Anchors	Response
1) Have you smoked at least 100 traditional cigarettes (commercial or hand-rolled) in your life?<br>N (%)	Yes	925 (18.1%)
	No *(go to section D)*	4164 (81.8%)
2) At what age did you start smoking traditional cigarettes?<br>Years, mean (min-max)		16 (11-38)
3) How many traditional cigarettes did you smoke per day?<br>mean (min-max)		6 (0-60)
4) How long have you been without smoking traditional cigarettes?	< 7 days	22 (2.4%)
	8 days-30 days	47 (5.1%)
	> 30 days-6 months	111 (12.21%)
	>6 months-1year	134 (14.6%)
	>1 year	606 (65.9%)
5) Do you remember why you started smoking traditional cigarettes? (You can tick more than one box)<br>N (%)	Peer pressure / at parties / my friends smoked	587 (63.5%)
	Because I tried and I liked it / it gave me pleasure	449 (48.5%)
	Grown-up feeling / sense of empowerment	296 (32.0%)
	Influence from family / there were smokers in my family	58 (6.3%)
	Influence from my partner	57 (6.2%)
	Because I was feeling anxious / stressed	213 (23.0%)
	Because it made me feel confident	191 (20.3%)
	Other, specify_______________	0
6) How did you manage to quit smoking traditional cigarettes? (You can tick more than one box)<br>N(%)	Unconventional therapies (e.g. acupuncture, phytotherapy, etc.)	3 (0.3%)
	Support groups	2 (0.2%)
	Nicotine replacement therapy (e.g. patches, chewing gum, tablets, etc.)	13 (1.4%)
	Alone, without help	854 (92.3%)
	With the help of a doctor or other healthcare professional	3 (0.3%)
	Smoke-free advice centre	1 (0.1%)
	Smoke-free apps (on smartphones / tablets)	14 (1.5%)
	Other, specify_______________	88 (9.5%)
7) Why did you quit smoking traditional cigarettes? (You can tick more than one box.))<br>N(%)	Health concern	656 (70.9%)
	Concern for the health of those around me	154 (16.6%)
	To save money	331 (35.8%)
	To feel psychologically free	253 (27.4%)
	To improve my look	202 (21.8%)
	I did not like it	223 (24.1%)
	Other, specify_______________	122 (13.2%)

Table S1D. Answers from Section D of the questionnaire: Electronic cigarettes or HTP users (6588 responders to items D1, 237 responders to items D2-D9, 1107 responders to items D10-D11, 6581 to items and D12, 290 responders to item D13-D19, 609 responders to items D20-D21).

**Question**	**Anchors**	**Response**
1) Have you used electronic cigarettes (e-cigs) in the last week? N (%)	Yes	237 (3,6%)
	No, but I did in the past *(go to question D10)*	870 (13.2%)
	No, never have *(go to question D12)*	5481 (83.2%)
2) How long have you been using e-cigs?	< 7 days	13 (5.5%)
	8 days-30 days	19 (8.1%)
	31 days-6 months	41 (17.4%)
	> 6 months-1 year	58 (24.7%)
	> 1 year	104 (44.3%)
3) What kind of e-liquids do you mostly use? (You can tick more than one box) N (%)	With nicotine, no flavours	42 (17.7%)
	With nicotine and flavours	163 (68.8%)
	No nicotine, with flavours	38 (16.0%)
4) How many ml of liquids do you use per day? N (%)	<2	143 (61.1%)
	2-3	65 (27.8%)
	≥ 4	26 (11.1%)
5) Where do you usually use e-cigs? N (%)	Only outdoors	26 (11.1%)
	Only indoors (e.g. home, car, smoking areas of public places)	25 (10.7%)
	Both indoors and outdoors	183 (78.2%)
6) Where do you use your e-cig on campus? (You can tick more than one box) N (%)	I do not use it	88 (37.1%)
	Outdoors (e.g. courtyard, balconies, external stairs, porches)	134 (56.5%)
	In smoking areas	51 (21.5%)
	Indoors (e.g. hallways, washrooms, study areas)	8 (3.1%)
7) When you are with your family, you use e-cigs: N (%)	Openly	166 (70.9%)
	Secretly	68 (29.1%)
8) Have you ever been advised by a doctor or other healthcare professional to quit e-cigs?N (%)	Yes	28 (12.0%)
	No	179 (76.5%)
	I do not remember	27 (11.5%)
9) Are you going to stop using e-cigs in the next six months? N (%)	Yes	45 (19.2%)
	No	119 (50.9%)
	I don't know	70 (29.9%)
10) Why did you start using e-cigs? (You can tick more than one box) N (%)	I think it is less dangerous to my health as compared to smoking traditional cigarettes	378 (34.1%)
	As an alternative to traditional cigarettes	479 (43.3%)
	Trendy / out of curiosity	391 (35.3%)
	So that I could smoke where it is forbidden to smoke traditional cigarettes	120 (10.8%)
	As an aid to quit traditional cigarettes	343 (31.0%)
	Other, specify ___	70 (6.3%)
11) After you started using e-cigs: N (%)	I have given up traditional cigarettes	196 (17.8%)
	I have started or gone back to smoking traditional cigarettes	351 (31.9%)
	I have been smoking less traditional cigarettes	293 (26.6%)
	I have been smoking more traditional cigarettes	31 (2.8%)
	I did not smoke and still do not smoke traditional cigarettes	231 (21.0%)
12) Have you used HTPs in the last week (e.g.. IQOS)? 89	Yes	290 (4.4%)
	No, but I did in the past (go to question 20)	319 (4.8%)
	No, never have (go to Section E)	5972 (90.7%)
13) How long have you been using HTPs N (%)	< 7 days	27 (9.3%)
	8 days-30 days	20 (6.9%)
	31 days-6 months	72 (24.9%)
	> 6 months-1 year	55 (19.0%)
	> 1 year	115 (39.8%)
14) How many refills do you use per day? N (%)	< 1 -4	123 (42.6%)
	5-10	116 (40.1%)
	11-15	28 (9.7%)
	≥ 16	22 (7.6%)
15) Where do you usually use HTPs? N (%)	Only outdoors	53 (18.3%)
	Only indoors (e.g. home, car, smoking areas of public places)	213 (8.0%)
	Both indoors and outdoors	4 (73.7%)
16) Where do you use your HTPs on campus? (You can tick more than one box) N (%)	I do not use it	72 (24.8%)
	Outdoors (e.g. courtyard, balconies, external stairs, porches)	199 (68.6%)
	In smoking areas	64 (22.1%)
	Indoors (e.g. hallways, washrooms, study areas)	2 (0.7%)
17) When you are with your family, you use HTPs N (%)	Openly	183 (63.3%)
	Secretly	106 (36.7%)
18) Have you ever been advised by a doctor or healthcare professional to quit HTPs?	Yes	39 (13.5%)
	No	208 (72.0%)

N (%)	I do not remember	42 (14.5%)
19) Are you going to quit HTPs in the next six months? N (%)	Yes	52 (18.0%)
	No	134 (46.4%)
	I don't know	103 (35.6%)
20) Why did you start using HTPs? (You can tick more than one box) N (%)	I think it is less dangerous to my health as compared to smoking traditional cigarettes	265 (43.5%)
	As an alternative to traditional cigarettes	281 (46.1%)
	Trendy / out of curiosity	179 (29.4%)
	So that I could smoke where it is forbidden to smoke traditional cigarettes	65 (10.7%)
	As an aid to quit traditional cigarettes	180 (29.6%)
	Other, specify_______________	55 (9.0%)
21) After you started using HTPs: N (%)	I have given up traditional cigarettes	175 (28.8%)
	I have started or gone back to smoking traditional cigarettes	165 (27.2%)
	I have been smoking less traditional cigarettes	171 (28.2%)
	I have been smoking more traditional cigarettes	20 (3.3%)
	I did not smoke and still do not smoke traditional cigarettes	76 (12.5%)

Table S1E. Answers from Section E of the questionnaire: Passive smoking (6579 responders).

Question	Anchors		Response
1) Do you live with any smokers?<br>N (%)	Yes, and they smoke in my presence / at home		1516 (23.0%)
	Specify product(s) (You can tick more than one box.):	traditional cigarettes	1378 (90.9%)
		e-cigs or HTPs	340 (22.4%)
		other (pipe / cigar)	113 (7.5%)
	Yes, but they do not smoke in my presence / at home		753 (11.4%)
	No		4310 (65.6%)
2) Do you usually spend leisure time with smokers N (%)	Yes, and they smoke in my presence / at home		3850 (58.5%)
	Specify product(s) (You can tick more than one box.):	traditional cigarettes	3699 (96.1%)
		e-cigs or HTPs	1724 (44.8%)
		other (pipe / cigar)	299 (7.8%)
	Yes, but they do not smoke in my presence / at home		508 (7.7%)
	No		2219 (33.7%)
3) Over the last week, have you been exposed to passive smoking continuously (for at least 10 minutes)?<br>N (%)	Yes		2698 (41.0%)
	No *(go to question 5)*		3878 (59.0%)
4) Over the last week, where have you been exposed to passive smoking continuously (for at least 10 minutes)?<br>N (%)	Outdoors *(go to question 4A)*		1802 (66.8%)
	Indoors *(go to question 4B and then 4C)*		154 (5.7%)
	Both indoors and outdoors *(go to question 4B and then 4C)*		742 (27.5%)
4A) which product were you exposed to? (You can tick more than one box)<br>N (%)	Traditional cigarettes		1742 (96.7%)
	E-cigs or HTPs		625 (34.7%)
	Other (pipe / cigar)		94 (5.2%)
4B) Over the last week, where have you been exposed to passive smoking indoors? (You can tick more than one box.)<br>N (%)	At home		484 (54.0%)
	In the car		286 (31.9%)
	Other (public places, friends' houses, etc.)		599 (66.9%)
4C) Which product were you exposed to? (You can tick more than one box.)<br>N (%)	Traditional cigarettes		847 (94.5%)
	E-cigs or HTPs		303 (33.8%)
	Other (pipe / cigar)		62 (6.9%)
5) In your house, smoking traditional cigarettes:<br>N (%)	Is not allowed in any rooms		2786 (42.4%)
	Is allowed only in some rooms		514 (7.8%)
	Is allowed only outdoors (e.g. balcony / terrace / garden)		2907 (44.2%)
	Is allowed everywhere		367 (5.6%)
6) In your house, using e-cigs and HTPs:<br>N (%)	Is not allowed in any rooms		2785 (42.4%)
	Is allowed only in some rooms		611 (9.3%)
	Is allowed only outdoors (e.g. balcony / terrace / garden)		2195 (33.4%)
	Is allowed everywhere		983 (15.0%)

Table S1F. Answers from Section F of the questionnaire: Knowledge of smoking health related issues and role of healthcare professionals (6571 responders to questions F1-F15, 658 responders to questions F16-F23).

Question	Anchors	Response
1) Is active cigarette smoking bad for your health? N (%)	Yes	6521 (99.2%)
	Yes, but only in particular conditions (e.g. illness, pregnancy)	22 (0.3%)
	No	3 (<0.1%)
	I don’t know	25 (0.4%)
2) Is active e-cig smoking bad for your health? N (%)	Yes	4896 (74.5%)
	Yes, but only in particular conditions (e.g. illness, pregnancy)	146 (2,2%)
	No	96 (1.5%)
	I don’t know	1432 (21.8%)
3) Is active HTP smoking bad for your health? N (%)	Yes	5365 (81.6%)
	Yes, but only in particular conditions (e.g. illness, pregnancy)	86 (1.3%)
	No	27 (0.4%)
	I don’t know	1093 (16.6%)
4) E-cigs and HTPs, as compared to traditional cigarettes, are: N (%)	More harmful to the health of smokers	217 (3.3%)
	Equally harmful to the health of smokers	2075 (31.6%)
	Less harmful to the health of smokers	2752 (41.9%)
	I don't know	1527 (23.2%)
5) Do you think passive smoking from traditional cigarettes is harmful to the health of non-smokers? N (%)	Yes	6174 (94%)
	Yes, but only in particular conditions (e.g. illness, pregnancy)	278 (4,2%)
	No	44 (0.7%)
	I don’t know	75 (1,1%)
6) Do you think passive smoking from e-cigs or HTPs is harmful to the health of non-smokers? N (%)	Yes	3643 (55.4%)
	Yes, but only in particular conditions (e.g. illness, pregnancy)	453 (6.9%)
	No	609 (9.3%)
	I don’t know	1866 (28.4%)
7) What's the appeal of e-cigs and HTPs in your opinion? (You can tick more than one box) N (%)	They do not leave a persistent smell	3150 (47.7%)
	I think active smoking of these products is less dangerous to your health than traditional cigarette smoking	2103 (31.8%)
	I think passive smoking of these products is less dangerous to your health than traditional cigarette smoking	1330 (20.1%)
	They may be used where traditional cigarettes cannot be smoked	2904 (44.0%)
	You have different flavours	3351 (50.7%)
	They are trendy	3665 (55.7%)
	I am not familiar with these products	590 (8.9%)
	I don't know	514 (7.8%)
	Other, specify	204 (3,1%)
8) What are negative features of e-cigs and HTPS in your opinion? (You can tick more than one box) N (%)	They are too expensive	1725 (26.1%)
	There is no certainty about the long-term health effects of these products	3979 (60.2%)
	They may lead non-smokers to smoke	3674 (55.6%)
	They are difficult to find	99 (1,5%)
	They require maintenance / must be handled carefully	1360 (20.6%)
	I am not familiar with these products	822 (12.4%)
	I don't know	733 (11.1%)
	Other, specify_______________	184 (2.8%)
9) Which initiatives can help quit smoking or prevent young people from starting smoking in your opinion? (You can tick more than one box) N (%)	Increase in prices	3807 (57.6%)
	Informative advertising on the harm of smoking	2486 (37.6%)
	More information in schools on the harm of smoking	3613 (54.7%)
	Ban on smoking on TV / in movies	1032 (15.6%)
	More information on anti-smoking centers	1811 (27.4%)
	Extension of the smoking ban to outdoors (e.g. parks / gardens / bus stops)	3639 (55.1%)
	None of these	433 (6.6%)
	I don’t know	245 (3.7%)
	Other, specify_______________	310 (4.7%)
10) Has the Covid-19 epidemic changed your smoking habits?	No *(go to question 13)*	5402 (82.3%)
	Yes *(go to question 11)*	1164 (17.7%)
11) How has the Covid-19 epidemic changed your smoking habits?	I quitted smoking	242 (20.8%)
	I started smoking	76 (6.5%)
	I smoke less traditional cigarettes or use less e-cigs / HTPs	438 (37.6%)
	I smoke more traditional cigarettes or use more e-cigs / HTPs	309 (26.5%)
	I went from traditional cigarettes to e-cig/HTP	92 (7.9%)
	I went from e-cig/HTP to traditional cigarettes	7 (0.6%)
12) Why have you changed your smoking habits? (You can tick more than one box)	Concern for my health or those around me	356 (30.06%)
	Because I was feeling anxious / stressed	425 (36.5%)
	Financial reasons	137 (11.8%)
	I don’t know	202 (17.4%)
	Other, specify_______________	292 (25.1%)

13) Do healthcare professionals serve as role models for their patients and society in terms of smoking habits? N (%)	Yes	3634 (55.4%)
	No	1809 (27.6%)
	I don't know	1113 (17.0%)
14) Should healthcare professionals routinely advise their patients who smoke traditional cigarettes to quit smoking? N (%)	Yes	5612 (85.6%)
	No	432 (6.6%)
	I don't know	511 (7.8%)
15) Should healthcare professionals regularly advise their patients who use e-cigs and / or HTPs to stop using these products? N (%)	Yes	4638 (70.8%)
	No	521 (7.9%)
	I don't know	1396 (21.3%)
16) Should healthcare professionals receive specific training on smoking cessation techniques? N (%)	Yes	554 (84.5%)
	No	50 (7.6%)
	I don't know	52 (7.9%)
17) Do healthcare professionals have a role in giving advice or information on smoking cessation to patients? N (%)	Yes	615 (93.8%)
	No	16 (2.4%)
	I don't know	25 (3.8%)
18) Are healthcare professionals who smoke traditional cigarettes less likely to advise patients to quit smoking? N (%)	Yes	313 (47.8%)
	No	177 (27.0%)
	I don't know	165 (25.2%)
19) Are healthcare professionals who use other products (e-cigs and / or HTPs) less likely to advise patients to quit smoking? N (%)	Yes	274 (41.8%)
	No	183 (27.9%)
	I don't know	198 (30.2%)
20) During your university studies, did you learn that it is important to record the history of tobacco use as part of a patient's general medical history? N (%)	Yes	546 (83.4%)
	No	63 (9.6%)
	I don't know	46 (7%)
21) During your university studies, have you ever received any formal training in smoking cessation approaches to be used with patients? N (%)	Yes	142 (21.7%)
	No	492 (75.1%)
	I don't know	21 (3.2%)
22) During which university programe, did you learn about the effects of cigarette smoking on health? (You can tick more than one box.) N (%)	During my Bachelor's degree program	239 (36.3%)
	During my Master's or single-cycle degree program	289 (43.9%)
	Never	109 (16.3%)
	I don't remember	54 (8.2%)
23) On which of the following topics concerning cigarette smoking would you be interested in receiving more information during your university studies? (You can tick more than one box) N (%)	Health effects of traditional cigarette smoking	260 (39.5%)
	Smoking cessation techniques	443 (67.3%)
	Health effects of passive smoking	364 (55.3%)
	Health effects of new products (e-cigs and / or HTPs)	491 (74.6%)
	Other, specify______________	11 (1,7%)
	I find my training on these topics satisfactory	55 (8.4%)

Table S1G. Answers from Section G of the questionnaire: Knowledge and attitudes towards Italian smoking legislation, and educational needs (6544 responders).

Question	Anchors	Response
1) To protect the health of non-smokers, the law prohibits smoking indoors in public places, e.g. restaurants, trains, etc. (Law no. 3 of 2003, art. 51). Do you think the law serves its purpose? N (%)	Yes	6527 (95.6%)
	No	199 (3.0)
	I don't know	88 (1,3%)
2) Are shock images on cigarette packs effective as a health warning? N (%)	Yes	1162 (17.8%)
	No	4567 (69.8%)
	I don't know	816 (12.5%)
3) Are you aware that it is forbidden to smoke in vehicles in the presence of children and pregnant women? N (%)	Yes	5142 (78.6%)
	No	1402 (21.4%)
4) Are you aware that it is forbidden to throw cigarette butts on the ground? N (%)	Yes	5789 (88.5%)
	No	755 (11.5%)
5) Are you aware of the damage caused by cigarette butts in the environment? N (%)	Yes	5977 (91.3%)
	No	566 (8.7%)
6) Are you aware that it is forbidden to smoke traditional cigarettes and e-cigs in the outdoor areas of schools and universities? N (%)	Yes	3117 (47.6%)
	No	3427 (52.4%)
7) Are you aware that it is forbidden to sell e-cigarettes with nicotine to minors (under 18)? N (%)	Yes	5574 (85.2%)
	No	970 (14.8%)
8) Are you aware that the University of Milan has anti-smoking regulations? N (%)	Yes	3233 (49.4%)
	No	3311 (50.6%)
9) In your opinion, is the ban on smoking traditional cigarettes in outdoors areas (e.g. courtyards, balconies, porches) of the University complied with? N (%)	Yes	206 (3.1%)
	No	4965 (75.9%)
	I don't know	1373 (21.0%)
10) In your opinion, is the ban on smoking e-cigarettes in outdoors areas (e.g. courtyards, balconies, porches) of the University complied with? N (%)	Yes	161 (2,5%)
	No	4796 (73.3%)
	I don't know	1587 (24.3%)
11) What initiatives could the University of Milan undertake to help smokers quit smoking and protect the health of non-smokers? (You can tick more than one box.) N (%)	Informative campaigns on the harm of smoking	2064 (39.8%)
	Greater control over compliance with existing bans	4166 (63.7%)
	Launching specific courses on smoking issues	1627 (24.9%)
	Offering smokers courses to help them quit smoking	3560 (54.4%)
	Other, specify_______________	443 (6.8%)

Table S2. Awareness of health issues related to active smoking (6571 responders).

	Is active smoking bad for your health?											
	Traditional cigarette				e-cig				HTP			
	**Yes**	**Yes, but only in particular conditions**	**No**	**I don't know**	**Yes**	**Yes, but only in particular conditions**	**No**	**I don't know**	**Yes**	**Yes, but only in particular conditions**	**No**	**I don't know**
**All subjects, N (%)**	6521 (99.2)	22 (<1)	3 (<1)	25 (0.4)	4896 (74.5)	146 (2.2)	96 (1.5)	1432 (21.8)	5365 (81.6)	86 (1.3)	27 (0.4)	10963 (16.6)
**Law, N (%)**	431 (98.9)	2 (<1)	1 (<1)	2 (<1)	347 (79.6)[F]	6 (1.4)	4 (<1)	79 (18.1)	363 (83.3)	6 (1.4)	1 (<1)	66 (15.1)
**PESS, N (%)**	865 (97.9)	9 (<1)	1 (<1)	9 (1.0)	674 (76.2)[F]	27 (3.1)	15 (1.7)	168 (19.0)	742 (83.9)[F]	18 (2.0)	1 (<1)	123 (13.9)
**Hum, N (%)**	1430 (99.2)	4 (0.3)	0 (<1)	7 (<1)	1038 (72.0)	35 (2.4)	22 (1.5)	346 (24.0)[D]	1159 (80.4)	18 (1.2)	4 (<1)	260 (18.0)[D]
**Med, N (%)**	966 (100.0)	0 (0.0)	0 (0.0)	0 (0.0)	799 (82.7)[B,C,F,G,H,I]	15 (1.6)	4 (<1)	148 (15.3)	848 (87.8)[C,F,H,I]	6 (<1)	4 (<1)	108 (11.2)
**Pha, N (%)**	482 (100.0)	0 (0.0)	0 (0.0)	0 (0.0)	371 (77.0)	13 (2.7)	5 (1.0)	93 (19.3)	404 (83.8)	7 (1.5)	3 (<1)	68 (14.1)
**STE, N (%)**	1341 (99.4)	3 (<1)	0 (0.0)	5 (<1)	938 (69.6)	34 (2.5)	32 (2.4)[D]	344 (25.5)[B,D]	1039 (77.0)	18 (1.3)	8 (<1)	284 (21.1)[B,D,E]
**Agr, N (%)**	346 (99.4)	0 (0.0)	1 (<1)	1 (<1)	257 (73.9)	3 (<1)	4 (1.1)	84 (24.1)[D]	287 (82.5)	4 (1.1)	1 (<1)	56 (16.1)
**Vet, N (%)**	168 (98.8)	2 (1.2)	0 (0.0)	0 (0.0)	115 (67.6)	2 (1.2)	4 (2.4)	49 (28.8)[D]	126 (74.1)	2 (1.2)	2 (1.2)	40 (23.5)[D]
**LMIC, N (%)**	396 (99.2)	2 (<1)	0 (0.0)	1 (<1)	281 (70.4)	10 (2.5)	5 (1.3)	103 (25.8)	314 (78.7)	7 (1.8)	3 (<1)	75 (18.8)[D]
**Sport, N (%)**	96 (100.0)	0 (0.0)	0 (0.0)	0 (0.0)	76 (79.2)	1 (1.0)	1 (1.0)	18 (18.8)	83 (86.5)	0 (0.0)	0 (0.0)	13 (13.5)
p	<0.003				<0.001				<0.001			

[a]= vs. no; [A]= higher than Law, [B]= higher than PESS, [C]=higher than Hum, [D]=higher than Med, [E]= higher than Pha, [F]= higher than STE, [G]= higher than Agr, [H]= higher than Vet, [I]= higher than LMIC

Table S3. Awareness of health issues related to passive smoking (6571 responders).

	**Is passive smoking bad for your health?**							
	**Traditional cigarette**				**e-cig/HTP**			
	**Yes**	**Yes. but only in particular conditions**	**No**	**I don't know**	**Yes**	**Yes, but only in particular conditions**	**No**	**I don't know**
**All subjects. N (%)**	6174(94)	278 (4.2)	44 (<1)	75 (1.1)	3643 (55.4)	453 (6.9)	609 (9.3)	1866 (28.4)
**Law. N (%)**	401 (92.0)	23 (5.3)[D]	4 (<1)	8 (1.8)	259 (59.4)	29 (6.7)	45 (10.3)	103 (23.6)
**PESS. N (%)**	813 (92.0)	51 (5.8)[D.E]	6 (<1)	14 (1.6)	479 (54.2)	70 (7.9)	98 (11.1)[D]	237 (26.8)
**Hum. N (%)**	1333 (92.5)	77 (5.3)[D]	11 (<1)	20 (1.4)	766 (53.2)	104 (7.2)	121 (8.4)	450 (31.2)[D]
**Med. N (%)**	943 (97.6)[A.B.C.F.G]	17 (1.8)	3 (<1)	3 (<1)	628 (65.0)[B.C.F.G.I]	44 (4.6)	61 (6.3)	233 (24.1)
**Pha. N (%)**	472 (97.9)[A.B.C.F.G]	9 (1.9)	0 (0.0)	1 (<1)	292 (60.6)[F.G]	26 (5.4)	46 (9.5)	118 (24.5)
**STE. N (%)**	1262 (93.6)	59 (4.4)[D]	10 (<1)	18 (1.3)	688 (51.0)	100 (7.4)	137 (10.2)	424 (31.4)[D]
**Agr. N (%)**	323 (92.8)	16 (4.6)	6 (1.7)	3 (<1)	171 (49.1)	27 (7.8)	39 (11.2)	111 (31.9)
**Vet. N (%)**	159 (93.5)	5 (2.9)	2 (1.2)	4 (2.4)	90 (52.9)	13 (7.6)	16 (9.4)	51 (30.0)
**LMIC. N (%)**	377 (94.5)	18 (4.5)	1 (<1)	3 (<1)	214 (53.6)	34 (8.5)	36 8 (9.0)	115 (28.8)
**Sport. N (%)**	91 (94.8)	3 (3.1)	1 (1.0)	1 (1.0)	56 (58.3)	6 (6.3)	10 (10.4)	24 (25.0)
p	<0.001				<0.001			

[a]= vs. no; [A]= higher than Law, [B]= higher than PESS, [C]=higher than Hum, [D]=higher than Med, [E]= higher than Pha, [F]= higher than STE, [G]= higher than Agr, [H]= higher than Vet, [I]= higher than LMIC

Table S4. Role of healthcare professionals (6571 responders).

	**Do healthcare professionals serve as role models for their patients and society in terms of smoking habits?**			**Should healthcare professionals advise patients to quit smoking?**			**Should healthcare professionals advise patients to quit vaping?**		
	**Yes N (%)**	**No N (%)**	**I don't know N (%)**	**Yes N (%)**	**No N (%)**	**I don't know N (%)**	**Yes N (%)**	**No N (%)**	**I don't know N (%)**
**All subjects**	3634 (55.4)	1809 (27.6)	1113 (17.0)	5612 (85.6)	432 (6.6)	511 (7.8)	4638 (70.8)	521 (7.9)	1396 (21.3)
**Law**	225 (51.6)	135 (31.0)[D]	76 (17.4)[D]	369 (84.6)	33 (7.6)[D]	34 (7.8)	318 (72.9)	34 (7.8)	84 (19.3)
**PESS**	423 (47.9)	277 (31.4)[D,E]	183 (20.7)[D,E]	735 (83.2)	67 (7.6)[D]	81 (9.2)[D]	606 (68.6)	73 (8.3)[D]	204 (23.1)[D]
**Hum**	684 (47.6)	468 (32.6)[D,E]	284 (19.8)[D,E]	1167 (81.3)	133 (9.3)[D,E]	135 (9.4)[D]	951 (66.3)	159 (11.1)[D,E]	325 (22.6)[D]
**Med**	720 (74.8)[A,B,C,E,F,G,H,I]	173 (18.0)	69 (7.2)	891 (92.6)[A,B,C,F,G,I]	32 (3.3)	39 (4.1)	778 (80.9)[A,B,C,F,G,I]	43 (4.5)	141 (14.7)
**Pha**	307 (63.7)[A,B,C,F,I]	112 (23.2)	63 (13.1)[D]	436 (90.5)[B,C,I]	18 (3.7)	28 (5.8)	364 (75.5)[C,F]	27 (5.6)	91 (18.9)
**STE**	725 (53.8)	372 (27.6)[D]	251 (18.6)[D]	1158 (85.9)	83 (6.2)	107 (7.9)[D]	906 (67.2)	110 (8.2)[D]	332 (24.6)[D]
**Agr**	187 (54.0)	95 (27.5)[D]	64 (18.5)[D]	294 (85.0)	22 (6.4)	30 (8.7)[D]	238 (68.8)	27 (7.8)	81 (23.4)[D]
**Vet**	95 (56.2)	46 (27.2)	28 (16.6)[D]	145 (85.8)	11 (6.5)	13 (7.7)	121 (71.6)	12 (7.1)	36 (21.3)
**LMIC**	202 (50.8)	115 (28.9)[D]	81 (20.4)[D]	328 (82.4)	30 (7.5)[D]	40 (10.1)[D]	279 (70.1)	32 (8.0)	87 (21.9)
**Sport**	66 (68.8)[B,C]	16 (16.7)	14 (14.6)	89 (92.7)	3 (3.1)	4 (4.2)	77 (80.2)	4 (4.2)	15 (15.6)
p	0.001			0.001			0.001		

[A]= higher than Law, [B]= higher than PESS, [C]=higher than Hum, [D]=higher than Med, [E]= higher than Pha, [F]= higher than STE, [G]= higher than Agr, [H]= higher than Vet, [I]= higher than LMIC
